# The Role of WWOX in Cancer Progression: Mechanisms and Therapeutic Potential

**DOI:** 10.3390/cancers17213435

**Published:** 2025-10-26

**Authors:** Huiyao Yang, Bin Liao, Juan Zhao, Yongsheng Li

**Affiliations:** 1 Department of Phase I Clinical Trial Ward, Chongqing Key Laboratory of Translational Research for Cancer Metastasis and Individualized Treatment, Chongqing University Cancer Hospital, Chongqing 400030, China; huiyaoyang1125@163.com (H.Y.); liaobin2011@163.com (B.L.); zjde2021@163.com (J.Z.); 2 Department of Medical Oncology, Chongqing University Cancer Hospital, Chongqing 400030, China

**Keywords:** WW domain-containing oxidoreductase, WWOX, tumor suppressor, cancer, therapeutic target

## Abstract

**Simple Summary:**

WW domain-containing oxidoreductase (WWOX), a tumor suppressor, is downregulated in various tumor tissues. It is correlated with tumorigenesis, progression, treatment resistance, and clinical prognosis. For instance, the downregulation of WWOX expression is associated with increased tumor grade and aggressiveness, along with lower recurrence-free and overall survival rates in patients diagnosed with Basal-like and Triple-Negative Breast Cancer. This review provides a comprehensive overview of the structure, function, and role of WWOX in tumors, emphasizing its critical regulatory effects on cancer. Additionally, it discusses the potential applications and future prospects of WWOX as a tumor biomarker and therapeutic target.

**Abstract:**

Discovered and cloned in 2000, the WW domain-containing oxidoreductase (*WWOX*) gene serves as a crucial tumor suppressor gene. Its expression is frequently downregulated in a wide spectrum of human malignancies, and this reduction is strongly correlated with accelerated tumor progression and poor patient prognosis. WWOX exerts its tumor-suppressive effects through direct physical interactions with numerous key signaling proteins. However, much of the current research remains in its early stages, particularly studies focusing on WWOX as a biomarker and WWOX-targeting therapies. Furthermore, there is a notable deficiency in related clinical validation, leading to uncertainties regarding clinical translation. This review specifically focuses on elucidating the significant contributions of WWOX in modulating critical oncogenic traits within cancer cells. We detail its impact on uncontrolled proliferation, invasive potential, metastatic spread, metabolic reprogramming that favors tumor growth, interactions with the immune response, and the maintenance of genetic stability. Following this exploration of WWOX’s diverse mechanistic roles in cancer biology, the review further discusses the emerging translational potential of targeting WWOX pathways, including its application as a prognostic biomarker and the development of strategies that exploit WWOX function or restoration for novel cancer therapeutics.

## 1. Introduction

**Figure 1:**
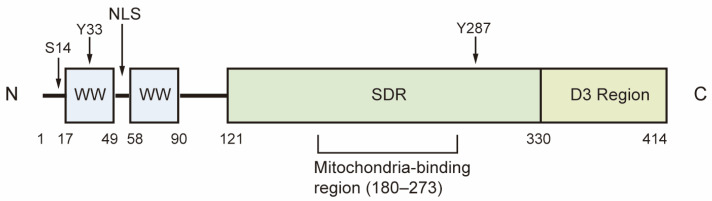
Structure of WWOX. The first WW domain of WWOX is responsible for its physical interactions with proteins. The SDR domain demonstrates redox activity, while the D3 region at the C-terminal is associated with pro-apoptotic activity. Ser14, Tyr33 and Tyr287 are the phosphorylation sites of WWOX. S14: Ser14; Y33: Tyr33; Y287: Tyr287; NLS: Nuclear localization signal; SDR, short-chain dehydrogenase/reductase.

WW domain-containing oxidoreductase (WWOX) is a highly conserved candidate tumor suppressor, with a homology of 93.9% between human and mouse WWOX proteins. The *WWOX* gene is located in the region of human chromosome 16q23.3-24.1, also known as *FRA16D*, which is recognized as the second most common chromosomal fragile site. This region is particularly susceptible to chromosomal breakage and rearrangement, leading to copy number variations in both germ and somatic cells [1,2]. The *WWOX* gene encodes a 46 kDa protein composed of 414 amino acids, featuring two WW domains at the N-terminal, a nuclear localization signal situated between the WW domains, and a short-chain dehydrogenase/reductase (SDR) domain at the C-terminal [1]. The WW1 domain interacts with proteins that contain the PPxY or LPxY motif, while the WW2 domain functions as a chaperone protein, enhancing the binding affinity of WW1. The SDR domain, characterized by its NSYK motif for binding both estrogen and androgen, demonstrates oxidoreductase activity and plays a crucial role in sex-steroid metabolism (Figure 1) [1,3].

**Figure 2:**
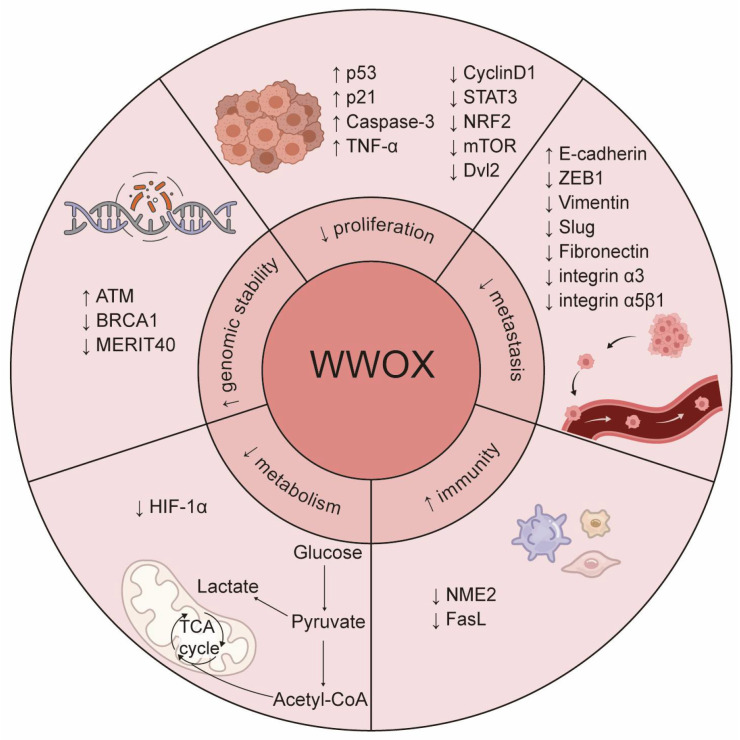
The roles of WWOX in cancer. WWOX functions as a tumor suppressor and critically regulates several cancer hallmarks, including the inhibition of proliferation and metastasis, prevention of immune evasion, regulation of metabolism, and maintenance of genomic stability. Specifically, WWOX modulates cell proliferation and survival by inhibiting the Wnt/β-catenin and JAK2/STAT3 signaling pathways. It activates the caspase cascade to induce apoptosis and inhibits metastasis by suppressing EMT. Furthermore, WWOX affects cell adhesion and migration by altering the expression of cell adhesion molecules, such as integrin α3 and integrin α5β1. Additionally, WWOX regulates the tumor microenvironment and supports the survival of immune cells, thereby preventing cancer cells from evading the immune system. It also regulates DNA repair pathways, contributing to genomic stability. Lastly, WWOX regulates tumor cell metabolism by modulating the metabolic reprogramming mediated by HIF-1α. The upward arrow signifies positive regulation, whereas the downward arrow denotes negative regulation.

WWOX is expressed at varying levels across different tissues and cell types, with particularly high expression observed in epithelial and nerve cells [4,5,6]. A decrease in WWOX expression has been linked to tumorigenesis and disease progression. Human germline biallelic mutations in *WWOX* are associated with spinocerebellar ataxia, autosomal recessive-12 (SCAR12), and WWOX-related epileptic encephalopathy (WOREE) syndrome [7,8,9]. These two debilitating neurological disorders typically present as severe epileptic encephalopathy and significant global developmental delay. Moreover, *Wwox*-null mice display similar intractable epilepsy, ataxia, hypomyelination, growth retardation, and premature death [8,10,11]. These mice also exhibit severe metabolic defects, including hypoglycemia, hypoalbuminemia, and abnormalities in steroid synthesis and bone metabolism, leading to mortality at approximately four weeks of age due to developmental delays and metabolic disorders [8,12,13,14]. Additionally, *Wwox*^−/−^ mice develop spontaneous tumors, with osteosarcoma detected histologically at postnatal days 3, 5, and 2.5 weeks, even in the absence of carcinogenic treatment. At an average age of 15 ± 1.5 months, *Wwox*^+/−^ mice show a significantly higher tumor burden and incidence compared to wild-type (WT) mice. Furthermore, treatment with ethyl nitrosourea (ENU), a chemical mutagen, markedly increases the incidence of lung cancer and lymphoma in *Wwox*^+/−^ mice [14]. Following ENU treatment, these mice develop tumors earlier than WT mice, with a significantly greater number of tumors observed per individual. Notably, *WWOX* exhibits loss of heterozygosity or reduced expression in tumors, which correlates with genomic instability, tumor progression, treatment resistance, and poor prognosis [2,14,15,16,17]. However, Watanabe et al. discovered that the levels of WWOX protein are elevated in non-invasive breast cancer and gastric cancer tissues, challenging the notion that WWOX functions as a classical tumor suppressor [5]. In contrast, WWOX is significantly downregulated during metastasis in these tissues. This tissue-specific heterogeneity in WWOX expression may be related to its functional roles across different types and subtypes of tumors or at various stages of tumor development, underscoring the necessity for further investigation. This article reviews the structure, function, and role of WWOX in tumors, while also discussing its potential applications and future prospects as a tumor biomarker and therapeutic target. Specifically, we summarize the effectors regulated by WWOX and their biological processes in tumors (Figure 2 and Table 1).

## 2. Epigenetic Regulation and Post-Translational Modification of WWOX

**Figure 3:**
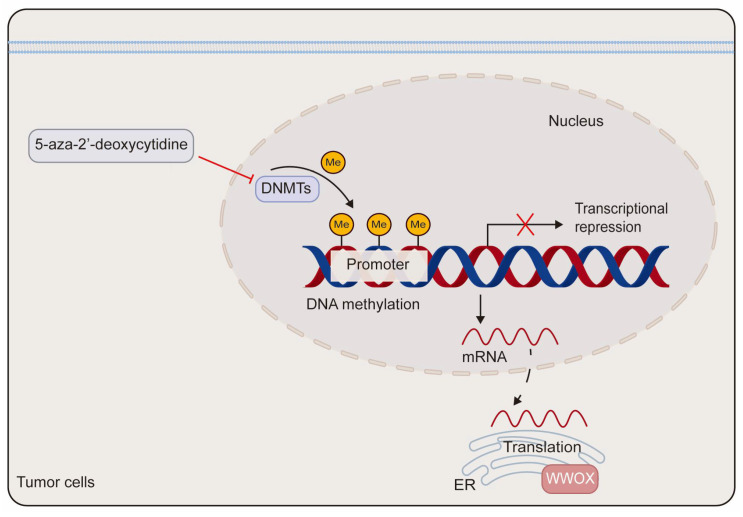
The schematic diagram of the epigenetic regulation of *WWOX*. The methylation of the *WWOX* promoter leads to the silencing of *WWOX* expression. This transcriptional silencing can be reversed by the DNA methyltransferase inhibitor 5-aza-2′-deoxycytidine. DNMTs refer to DNA methyltransferases.

DNA methylation has been implicated in the transcriptional silencing of *WWOX* in tumors. Research indicates that site-specific promoter hypermethylation of the *WWOX* gene is observed in 2 out of 15 (13%) primary pancreatic adenocarcinoma patients and in 2 out of 9 (22%) pancreatic cancer cell lines. Notably, *WWOX* expression is significantly elevated in the pancreatic cancer cell line Hs766T following treatment with the DNA methyltransferase inhibitor 5-aza-2′-deoxycytidine [54]. In comparison to adjacent non-neoplastic tissues, both the promoter and exon 1 regions of the *WWOX* gene exhibit hypermethylation in lung and breast cancers, which correlates with reduced *WWOX* expression [55]. Additionally, in prostate cancer-derived cells, DNA hypermethylation within the *WWOX* regulatory region contributes to the downregulation of *WWOX* expression. Treatment with either 5-aza-2′-deoxycytidine or trichostatin A, a histone deacetylase inhibitor, markedly enhances the expression of WWOX mRNA and protein in prostate cancer-derived cells (Figure 3) [23]. Furthermore, aberrant splicing of the *WWOX* gene contributes to the production of abnormal transcripts found in various cancers. This is supported by the identification of smaller spliced variants in breast and ovarian cancer cells, as well as by the high incidence of loss of heterozygosity and aberrant transcripts in esophageal, lung, and breast cancers [56,57].

The subcellular localization of WWOX varies depending on cell types and exogenous stimuli. Endogenous WWOX is predominantly found in the mitochondria and nucleus across various cell lines, while ectopic WWOX has also been detected in the Golgi apparatus [2,5,6,58,59]. The phosphorylation of WWOX influences its subcellular localization and functional roles. Stress or apoptotic stimuli induce the phosphorylation of WWOX at Tyr33, leading to its nuclear translocation and the subsequent induction of cell death [20,21,58,59]. Additionally, WWOX binds to estrogen and androgen through its SDR domain. Sex hormones trigger the phosphorylation of WWOX at Tyr33 and its nuclear translocation, independent of estrogen and androgen receptors [6]. Notably, the phosphorylation of WWOX at Tyr33 is essential for promoting cell death. When WWOX transitions from Tyr33 phosphorylation to Ser14 phosphorylation, it facilitates tumor progression and the development of Alzheimer’s disease [60]. The zinc finger-like protein that regulates apoptosis (Zfra) is a natural protein and one of WWOX’s binding partners. The artificially synthesized polypeptides Zfra1-31 and Zfra4-10 inhibit the growth of various tumors [60,61]. The Zfra peptide suppresses pulmonary metastasis of U87-MG glioblastoma cells by inhibiting WWOX Ser14 phosphorylation [61]. Treatment with the Zfra peptide significantly reduces the expression of pS14-WWOX in the lungs and brains of mice, thereby preventing the pulmonary metastasis of B16F10 melanoma cells and mitigating melanoma-induced neurodegeneration in the hippocampus, as well as the formation of cortical plaques. However, the Zfra peptide does not inhibit WWOX Tyr33 phosphorylation [60]. Furthermore, the pS14-WWOX7-21 peptide, which mimics the functional characteristics of endogenous pS14-WWOX, promotes melanoma growth in vivo and significantly inhibits the proliferation and death of 4T1 breast cancer cell spheroids mediated by ceritinib in vitro [62].

The intracellular tyrosine kinase, activated Cdc42-associated kinase (ACK1), promotes tumor progression in prostate cancer and hepatocellular carcinoma (HCC) by negatively regulating WWOX [63,64]. Specifically, activated ACK1 phosphorylates WWOX at Tyr287, which facilitates the polyubiquitination of WWOX and its subsequent degradation. While it is established that ACK1 is crucial for the degradation of WWOX, the specific ubiquitination site responsible for WWOX degradation and the ubiquitin E3 ligase involved in this process remain unclear [63].

Through mass spectrometry and protein interaction analysis, it has been demonstrated that WWOX physically binds to the ubiquitin E3 ligase ITCH via the WW1 domain. ITCH mediates the Lys63 linked ubiquitination of WWOX, which stabilizes WWOX and promotes its nuclear translocation, thereby enhancing cell death [65]. In addition, ITCH mediates the ubiquitination of WWOX at Lys274, facilitating WWOX’s nuclear translocation and its involvement in DNA damage repair [15].

## 3. WWOX in Cell Proliferation and Survival

WWOX inhibits cell proliferation and promotes apoptosis through various pathways. In prostate cancer, WWOX mediates G1 cell cycle arrest by downregulating cyclin D1 expression, which in turn suppresses the proliferation of 22Rv1 prostate cancer cells and inhibits tumor growth in nude mice [18]. Furthermore, in ovarian cancer, WWOX influences the cell cycle of ovarian cancer stem cells and inhibits their proliferation by downregulating the expression levels of cyclins, specifically cyclin E-CDK2 and cyclin D1-CDK4 [66]. Similarly, the overexpression of WWOX in osteosarcoma significantly enhances the expression of p53 and p21 while inhibiting cyclin D1 expression [19].

In addition, WWOX functions as a pro-apoptotic protein that promotes tumor cell death. Research has shown that the ectopic expression of WWOX triggers apoptosis via a caspase-dependent pathway and inhibits the growth of prostate, lung, breast, cervical cancer, and pancreatic cancer both in vitro and in vivo [23,24,25,26,27,28]. Moreover, the forced expression of WWOX in SNU387 cells not only induces spontaneous apoptosis but also suppresses cell proliferation. The JNK inhibitor SP600129 enhances WWOX-induced apoptosis and works synergistically to inhibit HCC growth alongside WWOX [67]. Additionally, the assembly of the WWOX/TIAF1/p53 ternary complex is crucial for the suppression of tumor progression. This complex functions by inhibiting metastasis and anchorage-independent growth, blocking Smad4-dependent SMAD promoter transactivation, and inducing apoptosis [68,69].

Stress stimuli, including tumor necrosis factor, ultraviolet light, and staurosporine, induce the conserved phosphorylation of Tyr33 in the WW1 domain of WWOX (denoted as pY33-WWOX). This phosphorylated form of WWOX interacts with various proteins independently of polyproline [20]. Notably, pY33-WWOX binds to Ser46-phosphorylated p53, and this interaction synergistically stabilizes p53, promoting apoptosis [20,21]. Additionally, the tyrosine kinase SRC phosphorylates WWOX at Tyr33, which facilitates its binding to the PPPY487 motif of p73, thereby enhancing WWOX-mediated apoptosis [29].

WWOX binds proteins through its WW1 domain, regulating their localization, stability, and transactivation functions, and participates in various signaling pathways, modulating cellular and biological processes such as tumor suppression, metabolism, and growth and development [20,35,47,70,71]. Notably, WWOX serves as a novel negative regulator of the Wnt/β-catenin pathway. It physically interacts with dishevelled2 (Dvl2), sequestering Dvl2 in the cytoplasm, which inhibits its function in stabilizing β-catenin and diminishes the transcriptional activity of the Wnt/β-catenin pathway [35,36]. Furthermore, BCL9-2 functions as a coactivator of β-catenin. In the nucleus, WWOX binds to BCL9-2, thereby inhibiting the Wnt/β-catenin signaling pathway [39]. In the context of triple-negative breast cancer, WWOX inhibits STAT3-dependent tumor cell growth and metastasis by modulating the IL-6/JAK2/STAT3 signaling pathway, leading to the downregulation of p-STAT3 target genes [33]. Co-immunoprecipitation studies have demonstrated that the WW1 domain of WWOX directly targets JAK2, inhibiting STAT3 activation by preventing JAK2 phosphorylation and its binding to STAT3. Furthermore, the WW1 domain of WWOX can specifically recognize and interact with STAT3, thereby inhibiting STAT3-mediated transcriptional activity of the IL-6 promoter, which results in reduced IL-6 mRNA expression and production [33]. Similarly, in hepatocellular carcinoma (HCC), toosendanin (TSN), a novel agonist of WWOX, dose-dependently increases WWOX mRNA and protein expression and enhances its interaction with STAT3 and Dvl2, thereby inhibiting both the JAK2/STAT3 and Wnt/β-catenin signaling pathways [34]. Given the interplay between WWOX and oncogenic signaling pathways, the application of Wnt/β-catenin inhibitors in WWOX-deficient contexts may demonstrate preclinical efficacy. Moreover, the combination of FDA-approved JAK/STAT inhibitors with strategies aimed at restoring WWOX function presents a promising direction for translational research. Furthermore, a notable association between WWOX and COTE1 has been observed. Overexpression of COTE1 in Focus HCC cells significantly reduces the expression of Tyr33 WWOX and Ser46 p53, thereby suppressing Tyr33 WWOX/Ser46 p53-mediated cell death [72]. Moreover, WWOX induces ferroptosis in HCC by mediating p53 activation and inhibiting NF-E2-related factor 2 (NRF2) through protein–protein interactions [22].

WWOX functions as a tumor suppressor by interacting with transcription factors and sequestering them in the cytoplasm, thereby inhibiting their transactivation functions. Aqeilan et al. demonstrated that WWOX physically binds to the PPPYFPPPY64 motif of Activator Protein-2γ (AP-2γ) and the PPPY64 motif of c-Jun through its first WW domain, leading to its sequestration in the cytoplasm and subsequent inhibition of its transcriptional activity and transactivation function [40,41]. Furthermore, they found that the WW1 domain of WWOX competes with Yes-associated protein (YAP) for binding to the PPIY1037 and PPPY1285 motifs of Erythroblastic Leukemia Viral Oncogene Homolog 4 (ErbB-4), thereby inhibiting YAP-mediated transactivation of ErbB-4 [42]. In osteosarcoma, WWOX binds to RUNX2 via the WW1 domain, thereby inhibiting its transactivation ability [43,44], whereas in breast cancer, the same domain facilitates the cytoplasmic sequestration of SMAD3, which suppresses its transcriptional activity [45]. Additionally, studies have shown that WWOX engages with SMAD3 and BMP2 to regulate TGFβ/BMP signaling, suppressing epithelial–mesenchymal transition (EMT) and cancer stem cell phenotypes, thereby inhibiting the progression of pancreatic ductal adenocarcinoma [46].

## 4. WWOX in Cell Invasion and Migration

During tumor progression and metastasis, the abnormal activation of EMT facilitates tumor cell migration and invasion, increases tumor stemness, and enhances resistance to chemotherapy and immunotherapy [73]. Key transcription factors, including SNAIL1/2, ZEB1/2, and TWIST1/2, are responsible for inducing EMT. Following EMT activation, the expression of the epithelial marker E-cadherin is suppressed, while mesenchymal markers such as N-cadherin, vimentin, and fibronectin are upregulated [73].

WWOX has been shown to regulate cell phenotype and inhibit metastasis by modulating epidermal and mesenchymal markers. The overexpression of WWOX in MG63 osteosarcoma cells significantly increases E-cadherin levels while reducing the expression of ZEB1, vimentin, and Slug, ultimately leading to the inhibition of cell proliferation, invasion, and migration [19]. Conversely, the loss of WWOX in HCC70 breast cancer cells results in decreased E-cadherin levels and increased fibronectin expression [37]. In MDA-MB-435s, HCC1806, and MDA-MB-231 cells, the restoration of WWOX elevates miR-146a expression, which directly targets the 3′-untranslated region (UTR) of fibronectin to inhibit its expression. The overexpression of WWOX in MDA-MB-231 cells significantly suppresses lung metastasis and colonization in immunocompromised mice, while also inhibiting fibronectin expression in vivo. Similarly, analysis of clinical data from The Cancer Genome Atlas-Breast Invasive Carcinoma (TCGA-BRCA) cohort reveals a positive correlation between WWOX expression and E-cadherin levels, alongside a negative trend with fibronectin levels in TNBC samples. Moreover, in MDA-MB-231 cells, doxycycline-induced WWOX expression leads to decreased levels of MYC and fibronectin. WWOX inhibits MYC activity by binding to MYC on chromatin, mitigating MYC’s suppression of miR-146a, which in turn inhibits fibronectin expression, EMT, tumor invasion, and metastasis [37]. Furthermore, miR-146a targets the 3′-UTR of *SMAD3*, resulting in reduced *SMAD3* expression and enhancing WWOX-mediated suppression of metastasis [36]. The classical Wnt signaling pathway orchestrates EMT, with Dvls serving as key molecules within this pathway. Phosphorylated Dvls recruit Axin to dismantle the β-catenin degradation complex, facilitating β-catenin stabilization and subsequent nuclear translocation. WWOX inhibits the Wnt/β-catenin signaling pathway and suppresses EMT by physically interacting with Dvl2 [36].

WWOX inhibits the tumorigenicity and metastasis of tumor cells by regulating their interaction with the extracellular matrix. As adhesion molecules, integrins facilitate the local peritoneal dissemination of ovarian cancer. Fibronectin, a primary ligand for integrin family members, is an extracellular matrix component that facilitates peritoneal metastasis. WWOX transfection significantly decreases the expression of membranous integrin α3 in PEO1 cells, as well as membranous integrin α3 and integrin α5β1 in SKOV3 cells, thereby inhibiting the adhesion of ovarian cancer cells to fibronectin and their migration [38]. Furthermore, WWOX expression promotes apoptosis in ovarian cancer cells cultured in suspension in vitro [38]. Additionally, WWOX regulates the migration of breast cancer and melanoma cells in vitro and inhibits metastasis in vivo by targeting p-STAT3 [33].

## 5. WWOX in the Immune Response

HCC is characterized by a high recurrence rate and poor prognosis. Immune checkpoint inhibitors (ICIs) have emerged as a significant treatment option for HCC. Research has demonstrated that WWOX deficiency induces an immunosuppressive tumor microenvironment, resulting in HCC resistance to PD-1 treatment. Liu et al. evaluated eight HCC tissues using cytometry by time-of-flight (CyTOF) analysis. The results shows that M2 macrophage markers, such as CD68 and CD204, are elevated in CD45^+^ cells of the WWOX^low^ subgroup, whereas no significant differences are observed in CD8^+^ T-cell associated markers, such as PD-L1, Granzyme B and PD-1. Notably, increased macrophage infiltration and decreased CD8^+^ T-cell infiltration have been observed in HCC tissues from patients deficient in WWOX [53]. Furthermore, higher levels of WWOX have been detected in the serum of patients who responded favorably to PD-1 antibody treatment. Immunohistochemical (IHC) results from 176 HCC patients indicate that WWOX is significantly upregulated in immune-responsive HCC tissues, with WWOX expression negatively correlated with CD68 and CD206 expression [53]. Prognostic analysis reveals that HCC patients with WWOX^low^CD68^high^ and WWOX^low^CD206^high^ tumors exhibit the poorest OS, and WWOX^low^CD206^high^ is an independent prognostic factor. In a WWOX^low^ humanized orthotopic HCC mouse model, PD-1 treatment significantly inhibited tumor growth in control mice, but not in the Huh7-shWWOX group. Additionally, CD8^+^ T-cell infiltration is reduced in the tumor tissues of Huh7-shWWOX mice [53]. Co-culturing WWOX^low^ HCC cells (HCCLM3-Vector and Huh7-shWWOX) with macrophages enhances macrophage recruitment, leading to significant upregulation of M2-like macrophage markers such as CD163, MRC1, IL-10, TGF-β, and ARG-1, along with increased CD206 protein expression. Oleic acid (OA), a product of fatty acid synthesis, promotes tumor progression by inducing the polarization of M2-like macrophages [53]. SCD5, a key enzyme in OA metabolism, shows upregulated protein and mRNA levels in Huh7-shWWOX cells, accompanied by increased OA concentration in the cell culture supernatant. Mechanistically, WWOX and KAT1 competitively interact with NME2 to inhibit its acetylation, thereby promoting NME2 binding to the SCD5 promoter and inhibiting SCD5 transcription, which in turn suppresses M2 macrophage polarization mediated by the SCD5-OA axis [53].

In addition, it was observed that the overexpression of WWOX in U251 human glioma cells significantly reduces the levels of FasL and TGF-β, promotes the proliferation of co-cultured Jurkat T cells, and decreases Jurkat T cell apoptosis. Conversely, WWOX deficiency impairs immune responses by inducing apoptotic signals mediated by Fas/FasL [30].

## 6. WWOX in Metabolism

WWOX is a key regulator of glucose metabolism. Studies in Drosophila have shown that WWOX binds to various proteins involved in metabolic processes, including isocitrate dehydrogenase, malate dehydrogenase, and Cu-Zn superoxide dismutase [74]. Notably, WWOX is not only crucial for glucose metabolism regulation but also exhibits expression changes in response to metabolic fluctuations. During the transition from glycolysis to oxidative phosphorylation, there is an increase in the steady-state levels of WWOX mRNA; conversely, under hypoxic conditions where cellular metabolism relies predominantly on glycolysis, WWOX mRNA levels decrease [75,76]. Metabolic reprogramming is a hallmark of cancer, with aerobic glycolysis representing the classical phenotype associated with this process. This phenotype is characterized by heightened glucose uptake and increased lactate production [77]. HIF-1α facilitates glycolysis by upregulating glucose transporter 1 (GLUT1) and transactivating glycolytic genes such as pyruvate kinase M2 (PKM2) and hexokinase 2 (HK2), thereby enhancing glucose uptake and utilization by cells [78].

Under normoxic conditions, mouse embryonic fibroblasts (MEFs) with a specific knockout of *Wwox* exhibit increased glucose uptake, elevated expression of HIF-1α, enhanced transcription of glycolytic genes, inhibition of the tricarboxylic acid cycle, decreased mitochondrial respiration, and increased glycolysis [47]. H-RAS transformed *Wwox* KO MEFs demonstrate greater tumorigenicity, characterized by higher levels of the HIF-1α target gene GLUT1 and augmented aerobic glycolysis compared to WT MEFs. The knockdown of WWOX in MCF7 breast cancer cells leads to the upregulation of HIF-1α glycolysis genes. Furthermore, the overexpression of WWOX in WWOX-depleted MCF7 cells suppresses the expression of HIF-1α target genes. The direct binding of the WWOX WW1 domain to HIF-1α impairs its transcriptional activity, consequently inhibiting its transactivation function. Additionally, WWOX facilitates HIF-1α hydroxylation and promotes its degradation. Wwox expression is downregulated in *Wwox* WT MEFs under hypoxic conditions or upon treatment with CoCl2. Overexpression of WWOX in *Wwox* WT MEFs under hypoxia leads to reduced expression of HIF-1α glycolysis target genes. Inhibition of HIF-1α expression using digoxin in *Wwox* KO MEFs reverses the abnormal increase in glucose uptake and suppresses the expression of HIF-1α target genes [47].

In addition, hepatocyte-specific *Wwox* knockout (*Wwox^ΔHep^*) mice exhibit elevated levels of Ki67 and hepatoma-related proliferation genes, including *c-Myc*, *c-Jun, c-Fos* and *Axin* in liver tissues following N-nitrosodiethylamine (DEN) treatment [48]. *Wwox^ΔHep^* mice demonstrate a higher incidence of HCC after DEN treatment compared to control mice. After 10 months of DEN treatment, *Wwox^ΔHep^* mice show a significant increase in HIF-1α protein levels and activity, alongside notable upregulation of PKM2 and GLUT1 protein expression [48]. Digoxin has been shown to inhibit HIF-1α transactivation, thereby suppressing tumor growth [79,80]. Following 6 months of DEN treatment, *Wwox^ΔHep^* mice were administered digoxin for an additional 8 months. Post-digoxin treatment, the expression of HIF-1α glycolysis target genes is downregulated, and liver tumor growth is significantly inhibited in DEN-*Wwox^ΔHep^* mice compared to those treated with saline [48]. These findings suggest that WWOX regulates HIF-1α-mediated metabolic reprogramming, thus suppressing tumor cell survival and growth. Importantly, either genetic or pharmacological depletion of HIF-1α can reverse the phenotypes associated with WWOX deficiency. The use of metabolic inhibitors, including HIF-1α inhibitors, digoxin, or GLUT1 inhibitors, may offer potential therapeutic strategies for treating WWOX-deficient tumors.

## 7. WWOX in Genomic Stability

**Figure 4:**
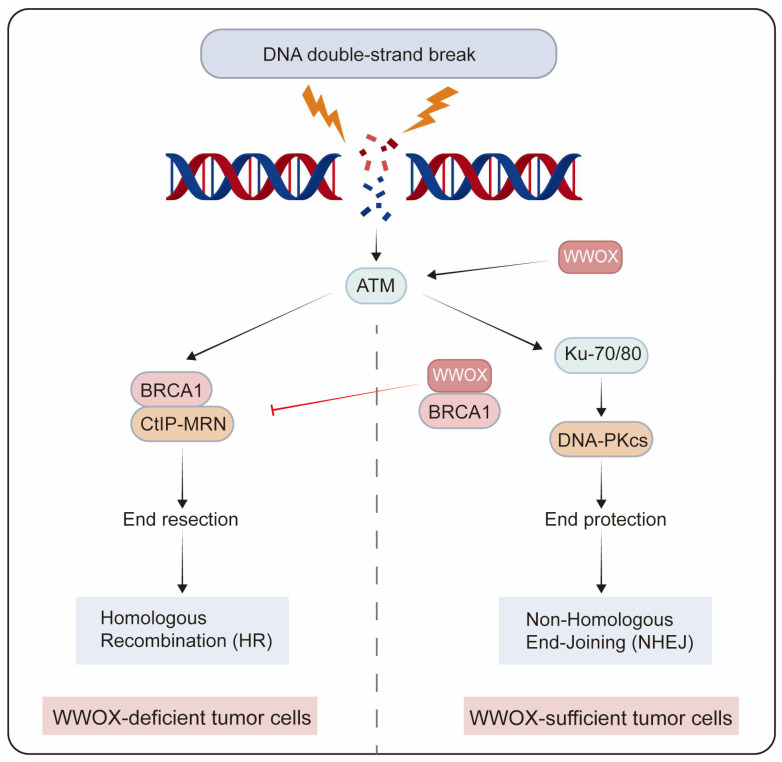
The role of WWOX in determining the choice of DSB repair pathways in tumor cells. WWOX-deficient breast cancer cells exhibit enhanced HR and reduced NHEJ repair. In contrast, in breast cancer cells with sufficient WWOX, the interaction between WWOX and BRCA1 inhibits HR while promoting NHEJ repair.

Genome integrity is crucial for normal cellular physiology. Following DNA damage, the activated DNA damage response plays a crucial role in maintaining genomic stability during replication by preventing potentially harmful mutations. DNA double-strand breaks (DSBs) represent one of the most severe types of DNA damage, which can result in chromosomal abnormalities. The two primary pathways for DSB repair are non-homologous end joining (NHEJ) and homologous recombination (HR) [81]. NHEJ directly rejoins the ends of DSBs, independent of sequence homology, while HR predominantly occurs during the S and G2 phases of the cell cycle, utilizing sister chromatids as templates for DSB repair. In the MCF7 breast cancer cell line, depletion of WWOX leads to an increase in DSBs following DNA damage induction, and the loss of the WWOX gene product results in genomic instability post-DNA damage. The activation of the DNA damage checkpoint kinase ataxia telangiectasia-mutated (ATM) is delayed in WWOX-deficient cells after DNA damage [15]. Moreover, WWOX-deficient breast cancer cells exhibit enhanced HR and reduced NHEJ repair, resulting in resistance to cisplatin and radiotherapy. BRCA1 is a pivotal factor in DNA end resection, a major determinant of HR. WWOX inhibits HR by interacting with Brca1 and promotes NHEJ repair [49]. Premature resection at DSBs causes dysregulated HR repair that is not confined to the S/G2 phases of the cell cycle, thereby increasing genomic mutations in cell populations resistant to IR and platinum treatment. Conversely, in MDA-MB231 cells that overexpress WWOX, the interaction between WWOX and BRCA1 predominantly facilitates the repair of DSBs through the NHEJ pathway [50]. Additionally, WWOX overexpression enhances NHEJ repair in BRCA1 wild-type TNBC cells (Figure 4) [51].

Low expression of WWOX is associated with poor response and shorter overall survival in cancer patients undergoing DNA damage induction therapy, such as radiotherapy or platinum-based treatments. WWOX expression may serve as a predictive biomarker for the efficacy of DNA damage induction therapy. The Mediator of RAP80 Interaction and Targeting 40 kDa protein (MERIT40) acts as an activator of HR and plays a crucial role in the DNA damage response (DDR). Hyperactivity of HR can lead to genomic instability, which is a contributing factor to cancer development. WWOX mitigates the excessive HR activity induced by MERIT40 overexpression by binding to the TBM2 motif of MERIT40, thereby hindering its interaction with Tankyrase [52]. In addition, WWOX promotes cellular senescence and maintains genome integrity in mouse embryonic fibroblasts (MEFs) by inhibiting the excessive production of reactive oxygen species (ROS). *Wwox* knockout MEFs exhibit reduced expression of p16^Ink4a^ and p21^Cip1/Waf1^ during serial passage in culture, leading to an escape from cellular senescence and increased genomic instability [82]. In summary, WWOX is pivotal in the selection of DNA repair pathways and the maintenance of genomic stability.

## 8. Therapeutic Potential of WWOX in Cancer

### 8.1. WWOX as a Potential Tumor Biomarker

*WWOX* genetic variants may serve as independent predictors of survival across multiple cancers. Low expression or deletion of *WWOX*, as well as copy number variation, have been associated with increased invasiveness and poor prognosis in various tumors, including lung cancer [83,84], breast cancer [33,85,86], HCC [87], gastric cancer [88], ovarian cancer [89], intrahepatic cholangiocarcinoma [90] and clear cell renal cell carcinoma [91] (Table 2). Research indicates that the depletion of *Wwox* and *p53* in mouse osteoblast progenitors accelerates osteosarcoma progression, resulting in a more aggressive form of the disease compared to *p53* knockout alone [92]. The loss of WWOX during the early stages of tumorigenesis promotes osteosarcomagenesis. Bone marrow cells from young non-osteosarcoma mice with a combined knockout of *Wwox* and *Trp53* exhibit significantly upregulated expression of Myc and its target MCM7, compared to bone marrow cells with *Trp53* knockout alone, demonstrating tumorigenicity both in vivo and in vitro [93].

Studies have demonstrated that breast cancer patients exhibiting low levels of WWOX expression tend to have a higher tumor grade, an increased risk of distant metastasis, and shorter recurrence-free survival and overall survival rates. This suggests that WWOX expression levels may serve as a predictive marker for the malignant progression of breast cancer [33]. Furthermore, while WWOX expression is generally low in HCC, it is significantly upregulated in the serum and tissues of HCC patients who respond immunologically to PD-1 treatment. WWOX deficiency promotes an immunosuppressive tumor microenvironment in HCC, thereby contributing to resistance to ICIs. Consequently, WWOX holds promise as a predictive biomarker for the efficacy of ICIs [53].

In addition, *WWOX* polymorphisms are associated with postoperative recurrence and tumor aggressiveness in HCC and prostate cancer [94,95]. Specifically, *WWOX* rs11644322 serves as a primary predictor for pancreatic cancer treated with gemcitabine, where the G > A single nucleotide polymorphism (SNP) in *WWOX* rs11644322 correlates with a poorer prognosis in pancreatic cancer patients [96]. Recently, Cheng et al. discovered that the G > T change at rs9922483 is associated with an elevated risk of mortality in gastric cancer patients, as evidenced by a genome-wide association study involving 796 patients in the eastern Chinese population [97]. The rs9922483 locus may interact long-range with the *WWOX* promoter, and the G > T alteration inhibits the transcriptional activity of this promoter. The T allele decreases the binding affinity of NR3C1, resulting in reduced *WWOX* expression, which subsequently lowers the survival rate of gastric cancer patients [97].

### 8.2. WWOX in Chemoresistance

**Figure 5:**
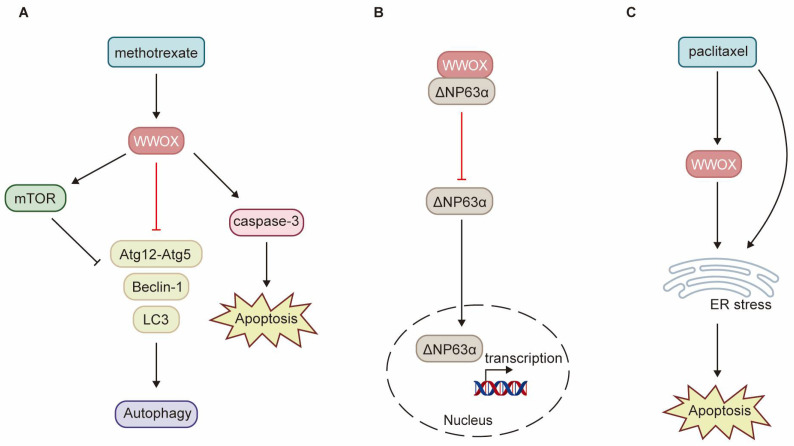
Schematic diagram illustrating the role of WWOX in chemoresistance. (**A**) In SCC, WWOX enhances the inhibition of autophagy mediated by mTOR, as well as apoptosis induced by methotrexate. (**B**) In osteosarcoma, WWOX mitigates chemoresistance mediated by ΔNp63α through direct interaction with ΔNp63α, effectively sequestering it within the cytoplasm. (**C**) WWOX facilitates apoptosis induced by ER stress, thereby augmenting cell death mediated by paclitaxel. ER refers to the endoplasmic reticulum.

WWOX enhances the sensitivity of cancer cells to chemotherapy through various pathways. Low levels of WWOX expression are frequently associated with tumor chemoresistance. Research has demonstrated that methotrexate (MTX) significantly upregulates WWOX protein expression, activates caspase-3, and induces apoptosis in squamous cell carcinoma (SCC) tumor tissues and SCC-4 and SCC-15 cell lines [32]. The induction of WWOX expression by MTX treatment is crucial for determining the sensitivity of SCC to MTX-induced apoptosis. Consequently, SCC-9 cells exhibit resistance to MTX due to the inability of MTX treatment to induce WWOX expression. By interacting with mTOR, WWOX enhances the MTX-induced phosphorylation of mTOR and p70S6K, inhibits the expression of autophagy-related proteins such as Beclin-1, Atg12-Atg5, and LC3-Ⅱ, and promotes MTX-induced apoptosis (Figure 5A) [32]. ΔNp63α, the most prevalent isoform of p63, can induce chemoresistance in tumor cells through multiple pathways. WWOX inhibits ITCH-mediated ubiquitination and degradation of ΔNp63α, thereby increasing its protein stability. Additionally, WWOX partners with ΔNp63α via the WW1 domain, sequestering ΔNp63α in the cytoplasm and inhibiting its transactivation function. Overexpression of WWOX has been shown to inhibit ΔNp63α-mediated resistance to cisplatin in SaOS2 osteosarcoma cells (Figure 5B) [98]. Paclitaxel exerts its antitumor effects by disrupting mitosis and activating endoplasmic reticulum (ER) stress. Notably, WWOX expression significantly enhances paclitaxel-mediated cell death in epithelial ovarian cancer (EOC) cells [16]. WWOX sensitizes EOC to paclitaxel through ER stress-induced apoptosis (Figure 5C). Furthermore, high WWOX expression is associated with longer overall survival and progression-free survival in ovarian cancer patients treated with paclitaxel [16]. However, there is currently a notable absence of clinical trials to validate these findings.

### 8.3. Compounds Related to WWOX

Given the high costs and elevated failure rates associated with new drug development, drug repurposing and the identification of new indications for existing drugs have emerged as promising strategies in the field of anti-tumor drug development. Recent studies on colorectal cancer have demonstrated that aspirin exerts an anti-tumor metastatic effect by inhibiting the TXA2-ARHGEF1 signaling pathway and enhancing T cell anti-tumor immunity [99]. Similarly, albendazole (ABZ), an anthelmintic agent, has been shown to dose-dependently upregulate WWOX expression and strengthen its interaction with p53 and C-MYC in HCC. This interaction leads to cell cycle arrest, induces apoptosis, and inhibits cell migration, indicating that ABZ may serve as a potential WWOX-targeted therapeutic agent for HCC [100].

Evodiamine and Toosendanin (TSN), both traditional Chinese medicinal compounds, have been identified as potential targeted agonists of WWOX. These compounds exert anti-tumor effects by dose-dependently upregulating the expression of WWOX [34,101,102]. Research indicates that TSN exhibits a strong binding affinity for WWOX. Both TSN alone and in combination with regorafenib can significantly enhance WWOX levels in vitro and in vivo, thereby suppressing the proliferation and migration of HCC cells [34,102].

In addition, WWOX regulates the ferroptosis induced by TSN. The activation of spermidine/spermine N1-acetyltransferase 1 (SAT1) facilitates ferroptosis by enhancing polyamine metabolism [103]. TSN markedly upregulates the protein expression of phosphorylated WWOX (p-WWOX) and phosphorylated p53 (p-p53) in MHCC-97L and HepG2 cells, enhancing the interaction between p-WWOX and p-p53 within the nucleus. This interaction leads to the upregulation of *SAT1* gene expression while downregulating *SLC40A1* gene expression [22]. Additionally, NF-E2-related factor 2 (NRF2) serves as the primary antioxidant transcription factor in the cellular response to oxidative stress, promoting the transcription of various protective target genes, including FPN1 and glutathione peroxidase 4 (GPX4) [104,105]. TSN enhances the interaction between WWOX and NRF2 as well as KEAP1 in MHCC-97L and HepG2 cells. The overexpression of WWOX leads to an increase in KEAP1 expression, inhibition of NRF2 expression and transcriptional activity, and subsequent downregulation of its downstream targets, FPN1 and GPX4 [22]. In summary, TSN modulates the transcriptional activity of p53 and NRF2 by regulating their interactions with WWOX, thereby influencing the expression of downstream ferroptosis-related target genes.

### 8.4. Other WWOX Modulators

The hydrogel drug delivery system exhibits excellent biocompatibility, stability, and sustained release capabilities, making it suitable for long-term and targeted treatment of tumors. Liu et al. transfected AY-27 bladder cancer cells with lentivirus (LV) to overexpress WWOX (LV-WWOX) and subsequently encapsulated this construct in gelatin hydrogel to create LV-WWOX-hydrogel (H-LV-WWOX) [31]. Research has demonstrated that lentivirus-mediated overexpression of WWOX significantly inhibits bladder tumor growth both in vitro and in vivo [31,106]. AY-27 cells treated with H-LV-WWOX and LV-WWOX show a marked reduction in cell viability, a significant increase in caspase-3 activity and expression levels, and elevated TNF-α expression [31]. In a rat orthotopic bladder cancer model (F344/AY-27), intravesical instillation of H-LV-WWOX promotes the generation of ROS, enhances caspase-3 activity, and increases TNF-α-mediated cell death [31]. The gene therapy mediated by the H-LV-WWOX drug delivery system achieves sustained release, high transduction efficiency, and reduced systemic toxicity, thereby offering a more effective and targeted therapeutic strategy for WWOX-based tumor treatment.

Chang et al. synthesized the WWOX7-21 and WWOX7-11 peptides to replicate the tumor-suppressive role of endogenous WWOX by mimicking its three-dimensional structures and generating specific antibodies [61,62]. Tail vein injections of either the WWOX7-21 or WWOX7-11 peptide significantly inhibited the growth and metastasis of melanoma cells in BALB/c mice. Furthermore, the WWOX7-21 peptide markedly enhanced ceritinib-mediated death in 4T1 breast cancer stem cell spheroids [62]. This study further validated the critical role of WWOX in inhibiting tumor growth and metastasis, indicating that both the WWOX7-21 and WWOX7-11 peptides hold promise for tumor inhibition in vivo. However, these studies are still in the early preclinical phase. The development of peptide-based drugs encounters several practical challenges, including poor stability, short half-life, and a protracted clinical development process. Transitioning from laboratory research to clinical application continues to pose significant challenges.

RNA-based therapies represent a novel approach to tumor treatment. Certain endogenous non-coding RNAs (ncRNAs) are capable of regulating the expression of tumor-related genes and are implicated in tumorigenesis and progression. Targeting these ncRNAs for the development of anti-tumor drugs shows significant promise [107]. Research has indicated that exosomal miR-625-3p, secreted by cancer-associated fibroblasts, promotes the proliferation, invasion, and EMT of CRC cells, inhibits apoptosis, and enhances chemotherapy resistance by downregulating CELF2/WWOX. Inhibition of miR-625-3p expression markedly reduces the malignant behaviors of CRC cells [108]. Moreover, miR-134 is significantly upregulated in head and neck squamous cell carcinoma (HNSCC) tissues, and its expression is negatively correlated with WWOX levels [109]. Reporter assays have demonstrated that in OECM-1 and SAS HNSCC cell lines, ectopically expressed miR-134 directly targets the 3′-UTR of *WWOX*, thereby inhibiting *WWOX* reporter activity and promoting cell proliferation, migration, and invasion. Furthermore, ectopic expression of WWOX significantly suppresses the invasion induced by miR-134 in HNSCC cells [109]. Additionally, long non-coding RNA WWOX-AS1 is downregulated in HCC and osteosarcoma patient tumor tissues and cell lines, with its overexpression significantly inhibiting the progression of both HCC and osteosarcoma [110,111]. Furthermore, WWOX-AS1 overexpression notably inhibits the proliferation, migration, and EMT of HepG2 and Huh7 cells, acting as an RNA sponge for miR-20b-5p, which in turn upregulates WWOX expression by sequestering miR-20b-5p [110]. Therefore, ncRNA-based therapies present promising strategies for tumor treatment by directly or indirectly enhancing WWOX expression, thereby inhibiting tumor progression.

## 9. Conclusions and Perspectives

**Figure 6:**
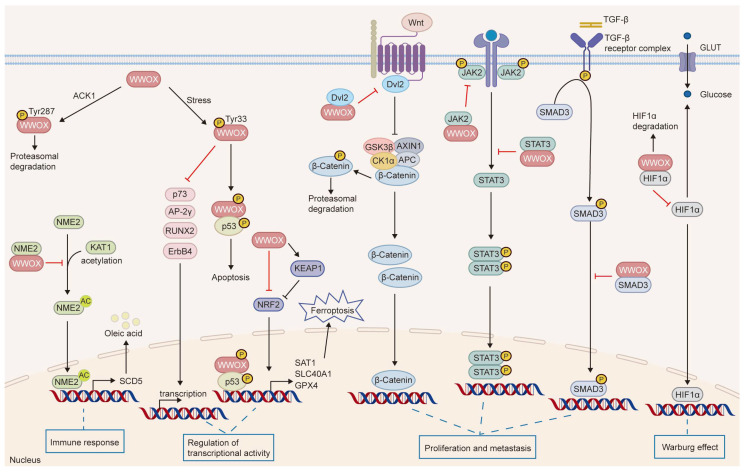
The signaling pathways influenced by WWOX. WWOX modulates several cellular pathways, including JAK2/STAT3, Wnt/β-catenin, TGFβ, and KEAP1/NRF2. It interacts with HIF-1α to inhibit its transactivation function. Furthermore, WWOX binds to p73, AP-2γ, RUNX2, and ErbB4, sequestering them in the cytoplasm, which inhibits their transcriptional and transactivation functions. Additionally, WWOX binds to NME2, inhibiting its acetylation and the transcription of SCD5, thereby suppressing M2 macrophage polarization mediated by the SCD5-OA axis. Moreover, the interaction between phosphorylated WWOX and phosphorylated p53 induces apoptosis.

WWOX is downregulated in various tumor tissues and is associated with tumorigenesis, progression, treatment resistance, and clinical prognosis, indicating its potential as a novel biomarker. As a tumor suppressor, WWOX regulates several critical processes in cancer, including cell proliferation, apoptosis, invasion, migration, genomic instability, metabolic reprogramming, and tumor immunity (Figure 6). Furthermore, WWOX is recognized as a systemic transcriptional regulator that engages in multiple cellular signaling pathways through protein–protein interactions. Notably, alterations in WWOX phosphorylation sites contribute to its diverse roles in tumors. Therefore, further investigation into the mechanisms by which WWOX operates in different tumor cell types is essential.

Future research should prioritize a comprehensive validation of WWOX as a biomarker in clinical data, which holds significant implications for tumor diagnosis and prognosis assessment. Restoring WWOX expression in tumors has been shown to effectively inhibit tumor growth, thereby positioning WWOX as a promising target for anti-tumor therapeutic strategies, despite the inherent challenges. Current animal experiments have investigated various WWOX-targeting drugs, including small molecule compounds, peptides, and RNA-based therapies. However, further clinical trials are essential to evaluate their safety and efficacy. Additionally, the discovery and design of WWOX activators could facilitate the development of more effective anti-tumor therapies, although the development and testing process is often lengthy and costly. Furthermore, WWOX may exhibit varying expression levels across different types and subtypes of tumors or at distinct stages of tumor development, necessitating further investigation into the tissue-specific differences in WWOX function.

## Figures and Tables

**Table 1:** Downstream effectors of WWOX.

Downstream Effectors	Regulation Form	Effect of Regulation	Biological Process	Related Cancer	Ref
Cyclin D1	alters cyclin D1 expression	decreases cyclin D1 expression	cell cycle, cell proliferation	Prostate cancer, osteosarcoma	[18,19]
p53	alters p53 expression, binding	increases p53 activity and expression	cell cycle, apoptosis, ferroptosis	Osteosarcoma, hepatocellular carcinoma	[19,20,21,22]
p21	alters p21 expression	increases p21 expression	cell cycle	Osteosarcoma	[19]
caspase-3	alters caspase-3 expression	increases caspase-3 activity and expression	apoptosis	Prostate cancer, lung cancer, breast cancer, cervical cancer, pancreatic cancer	[23,24,25,26,27,28]
p73	binding	sequesters p73 in cytoplasm, inhibits p73-mediated transcription and transactivation	apoptosis	Breast cancer, osteosarcoma	[29]
FasL	alters FasL expression	reduces the level of FasL	apoptosis	Glioma	[30]
TNF-α	alters TNF-α expression	increases TNF-α expression	apoptosis	Bladder cancer	[31]
mTOR	binding	promotes apoptosis, inhibits autophagy	apoptosis, autophagy	Squamous cell carcinoma	[32]
NRF2	binding	decreases NRF2 expression, inhibits NRF2-mediated transcription	ferroptosis	Hepatocellular carcinoma	[22]
STAT3	binding	inhibits JAK2/STAT3 signaling pathway	cell growth, cell migration, metastasis	Breast cancer, melanoma	[33,34]
Dvl2	binding	inhibits Wnt/β-catenin signaling pathway	EMT, cell growth	Breast cancer, hepatocellular carcinoma	[34,35,36]
E-cadherin	alters E-cadherin expression	increases E-cadherin expression	EMT, cell proliferation, invasion, migration	Osteosarcoma, breast cancer	[19,37]
ZEB1	alters ZEB1 expression	decreases ZEB1 expression	EMT, cell proliferation, invasion, migration	Osteosarcoma	[19]
Vimentin	alters vimentin expression	decreases vimentin expression	EMT, cell proliferation, invasion, migration	Osteosarcoma	[19]
Slug	alters Slug expression	decreases Slug expression	EMT, cell proliferation, invasion, migration	Osteosarcoma	[19]
Fibronectin	alters fibronectin expression	decreases fibronectin expression	EMT, metastasis	Breast cancer	[37]
MYC	binding	inhibits MYC activity, decreases MYC expression	EMT, metastasis, invasion	Breast cancer	[37]
integrin α3	alters integrin α3 expression	decreases integrin α3 expression	cell migration	Ovarian cancer	[38]
integrin α5β1	alters integrin α5β1 expression	decreases integrin α5β1 expression	cell migration	Ovarian cancer	[38]
BCL9-2	binding	inhibits BCL9-2-mediated transcription, inhibits Wnt/β-catenin signaling pathway	transcription regulation	Breast cancer	[39]
AP-2γ	binding	inhibits AP-2γ-mediated transcription and transactivation	transcription regulation	Breast cancer	[40]
c-Jun	binding	inhibits c-Jun-mediated transcription and transactivation	transcription regulation	Cervical cancer	[41]
ErbB-4	binding	inhibits ErbB-4-mediated transactivation	transcription regulation	Breast cancer	[42]
RUNX2	binding	inhibits RUNX2-mediated transactivation	transcription regulation	Osteosarcoma	[43,44]
SMAD3	binding	inhibits SMAD3-mediated transcription, attenuates TGF-β signaling pathway	transcription regulation	Breast cancer, pancreatic ductal adenocarcinoma	[45,46]
HIF-1α	binding	inhibits HIF-1α-mediated transactivation, promotes HIF-1α degradation	transcription regulation, aerobic glycolysis	Breast cancer, hepatocellular carcinoma	[47,48]
ATM	binding	activates ATM	DNA damage repair	Breast cancer	[15]
BRCA1	binding	inhibits HR, promotes NHEJ repair	DNA damage repair	Breast cancer	[49,50,51]
MERIT40	binding	mitigates excessive HR activity	DNA damage repair	Breast cancer	[52]
NME2	binding	inhibits NME2 acetylation	immune responses	Hepatocellular carcinoma	[53]

**Table 2:** WWOX is a biomarker for prognostication and predicting drug sensitivity in various types of cancer.

Cancer	Expression or Methylation Status	Clinical Indicator	Relevance	Independent or Not	Evidence	Ref.
Non-small cell lung cancer	methylation status	Poor prognosis	Negative	Not mentioned	Clinical	[84]
Triple-negative breast cancer	expression	RFS, OS	Positive	Independent	Clinical	[33,37]
Basal-like breast cancer	expression	DFS	Positive	Not	Clinical	[86]
Hepatocellular carcinoma	expression	RFS, OS	positive	Independent	Clinical	[87]
Epithelial ovarian cancer	expression	OS, PFS, response to paclitaxel	Positive, high WWOX expression-better response	Independent	Clinical	[16]
Gastric adenocarcinoma	expression	OS	Positive	Independent	Clinical	[88]
Intrahepatic cholangiocarcinoma	methylation status	OS, cumulative recurrence	Positive, negative	Independent	Clinical	[90]
Clear cell renal cell carcinoma	expression	Poor prognosis	Negative	Not	Clinical	[91]

## Abbreviations

WWOX	WW domain-containing oxidoreductase
SDR	Short-chain dehydrogenase/reductase
SCAR12	Spinocerebellar ataxia, autosomal recessive-12
WOREE	WWOX-related epileptic encephalopathy
WT	Wild-type
ENU	Ethyl nitrosourea
Zfra	Zinc finger-like protein that regulates apoptosis
ACK1	Activated Cdc42-associated kinase
HCC	Hepatocellular carcinoma
Dvl2	Dishevelled2
TSN	Toosendanin
NRF2	NF-E2-related factor 2
AP-2γ	Activator Protein-2γ
YAP	Yes-associated protein
ErbB-4	Erythroblastic Leukemia Viral Oncogene Homolog 4
EMT	Epithelial–mesenchymal transition
ICIs	Immune checkpoint inhibitors
IHC	Immunohistochemical
GLUT1	Glucose transporter 1
PKM2	Pyruvate kinase M2
MEFs	Mouse embryonic fibroblasts
DEN	N-nitrosodiethylamine
DSBs	DNA double-strand breaks
NHEJ	Non-homologous end joining
HR	Homologous recombination
ATM	Ataxia telangiectasia-mutated
MERIT40	Mediator of RAP80 Interaction and Targeting 40 kDa protein
ROS	Reactive oxygen species
MTX	Methotrexate
SCC	Squamous cell carcinoma
ER	Endoplasmic reticulum
EOC	Epithelial ovarian cancer
ABZ	Albendazole
SAT1	Spermidine/spermine N1-acetyltransferase 1
FPN1	Ferroportin 1
GPX4	Glutathione peroxidase 4
HNSCC	Head and neck squamous cell carcinoma
